# Universals and cultural diversity in the expression of gratitude

**DOI:** 10.1098/rsos.180391

**Published:** 2018-05-23

**Authors:** Simeon Floyd, Giovanni Rossi, Julija Baranova, Joe Blythe, Mark Dingemanse, Kobin H. Kendrick, Jörg Zinken, N. J. Enfield

**Affiliations:** 1 Department of Anthropology, Universidad San Francisco de Quito, Diego de Robles, Quito 170157, Ecuador; 2 Language and Cognition Department, Max Planck Institute for Psycholinguistics, Wundtlaan 1, Nijmegen 6525XD, The Netherlands; 3 Department of Finnish, Finno-Ugrian, and Scandinavian Studies, University of Helsinki, Vuorikatu 3A, Helsinki 00100, Finland; 4 Department of Linguistics, Macquarie University, Macquarie Walk, North Ryde, Sydney, NSW 2109, Australia; 5 Department of Language and Linguistic Science, University of York, Heslington, York YO10 5DD, UK; 6 Department of Pragmatics, Institute for the German Language in Mannheim, R5 6-13, Mannheim 68161, Germany; 7 Department of Linguistics, The University of Sydney, John Woolley Building A20, Science Road, Sydney, NSW 2006, Australia

**Keywords:** gratitude, reciprocity, assistance, collaboration, social interaction, cross-cultural

## Abstract

Gratitude is argued to have evolved to motivate and maintain social reciprocity among people, and to be linked to a wide range of positive effects—social, psychological and even physical. But is socially reciprocal behaviour dependent on the expression of gratitude, for example by saying ‘thank you’ as in English? Current research has not included cross-cultural elements, and has tended to conflate gratitude as an emotion with gratitude as a linguistic practice, as might appear to be the case in English. Here, we ask to what extent people express gratitude in different societies by focusing on episodes of everyday life where someone seeks and obtains a good, service or support from another, comparing these episodes across eight languages from five continents. We find that expressions of gratitude in these episodes are remarkably rare, suggesting that social reciprocity in everyday life relies on tacit understandings of rights and duties surrounding mutual assistance and collaboration. At the same time, we also find minor cross-cultural variation, with slightly higher rates in Western European languages English and Italian, showing that universal tendencies of social reciprocity should not be equated with more culturally variable practices of expressing gratitude. Our study complements previous experimental and culture-specific research on gratitude with a systematic comparison of audiovisual corpora of naturally occurring social interaction from different cultures from around the world.

## 1. Introduction

Social reciprocity is a basic element of human organization that involves the mutual exchange of goods, services and support among individuals, allowing for the distribution and augmentation of human agency in ways that individuals could not achieve alone. People can expect others to assist them in the achievement of individual goals (e.g. getting the salt from the opposite side of the table) as well as to collaborate in the achievement of common goals (e.g. removing a fallen branch from the middle of the road), and they can anticipate that others will expect analogous forms of assistance and collaboration in return. This reflects principles of cooperation that have been argued to be at the centre of human evolution [1], including the development of human communication [2,3].

A number of studies have suggested that the ability of individuals to experience gratitude to others is an important feature of human cognition, and is key to motivating and maintaining social reciprocity [4–8]. Other studies have also suggested that the experience of gratitude is linked to a wide range of positive effects on human well-being, including improved psychological and physical health, better relationships, less aggression, more self-esteem and even better sleep [9–11]. These findings are typically taken to imply that gratitude's positive effects depend on people verbally expressing it [12–14], for example by saying ‘thank you’. This has led popular science news outlets to make statements like: ‘According to positive psychologists, the words ‘thank you’ are no longer just good manners, they are also beneficial to the self’ [15], and similar conclusions have gained currency in self-help books [16]. These conclusions and the research that motivates them, however, are primarily based on English-speaking society, and often on the experience and behaviour of individuals as measured in the constrained contexts of laboratories.

**Figure 1.**
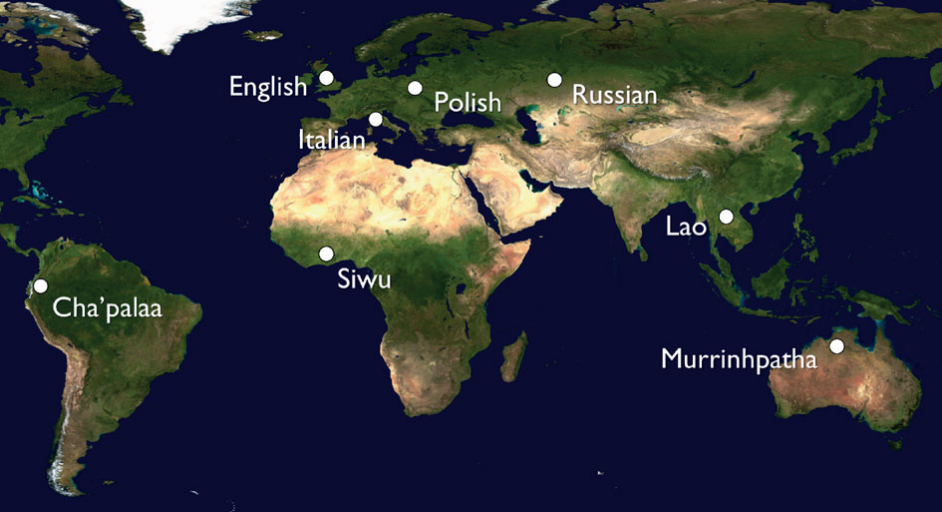
World map showing locations of data collection for the eight languages involved in the study. (Credit: satellite composition of the Earth's surface by NASA.)

We argue that, in order to better understand the role of gratitude in the maintenance of social reciprocity, we need to extend the investigation beyond English-speaking society. Also, we need to differentiate gratitude as an *emotion* from gratitude as a *linguistic practice*, which is better observed in the ecological validity of everyday social interaction rather than under artificial conditions in a laboratory*.* The study presented here asks to what extent people express gratitude for another's assistance or collaboration in everyday life across a diverse sample of languages from five continents (figure 1). If gratitude's role in the maintenance of social reciprocity in different societies is evidenced by its verbalization (e.g. ‘thank you’), we would expect this to occur frequently in episodes of informal everyday interaction where someone seeks and obtains a good, service or support from another. What we find instead is that expressions of gratitude in these episodes are remarkably rare, suggesting that social reciprocity relies to a large extent on tacit understandings of people's rights and duties surrounding mutual assistance and collaboration. At the same time, we also find minor cross-cultural variation, with slightly higher rates in Western European languages, including English, showing that the potentially universal experience of gratitude should not be conflated with culturally variable practices of expressing gratitude.

The idea that gratitude's role in social reciprocity is tied to saying ‘thank you’ appears to be largely based not on systematic empirical observation but on attitudes about politeness in English-speaking society. This is evidenced, among other things, by how English-speaking parents socialize children to politeness routines [17–19] and by the hundreds of English books and websites dedicated to teaching children to say ‘thank you’ (e.g. *The Berenstain Bears Say Please and Thank You* [20]), which do not exist in such great numbers, if at all, for most other languages. Different cultures have different styles of language socialization [21], and the expression of gratitude by parents to children is said to be rare in many societies (e.g. in Chinese mother–child interaction [22]). Second-language learners of English report difficulties adapting to frequently saying ‘thank you’ [23–25], and studies of other cultures show that thanking is frequently considered bizarre or rude [26,27]. Our study contributes to this line of research by demonstrating that social and prescriptive attitudes about politeness like those found among English speakers may not be reflected in people's actual behaviour. We find that, in informal everyday interaction across the world, the general norm is to tacitly acknowledge another's cooperative behaviour without explicitly saying ‘thank you’, but by simply continuing with one's activities, relying on a shared understanding of the good, service or support received as part of a system of social rights and duties governing mutual assistance and collaboration.

## 2. Material and methods: overview

The link between gratitude and social reciprocity has been extensively studied in controlled laboratory contexts, with only few empirical studies of gratitude **‘**in the wild’, that is, in ecologically valid contexts of everyday life. Other methods like self-report questionnaires often do not reliably reflect actual behaviour [28,29], and the few studies based on naturally occurring interaction have tended to focus on just one language (typically English) usually in a specific setting (e.g. the library), leaving questions about the generalizability and cross-cultural validity of the findings [30,31]. To be able to study gratitude in a wider range of settings, and to do so across a diverse set of cultures, we need audiovisual recordings of naturally occurring informal social interaction from around the world. Methods and techniques for obtaining and analysing cross-culturally comparable data from informal social interaction are only recent developments. A key part of this process is identifying comparable episodes of everyday life across different speakers, settings and societies. For a study of gratitude, the most relevant circumstance of social interaction is when someone seeks and obtains another's cooperation. These recurrent exchanges of requests and responses provide a natural control that allows for comparing similar interactional sequences across different languages and cultures [32]. In this case, every request (e.g. ‘Can you pass the salt?’) that is complied with (e.g. by passing the salt) creates an opportunity to express gratitude (e.g. by saying ‘Thanks’). We identified and sampled such sequences in audiovisual corpora of informal everyday interaction in eight languages. Focusing on everyday household and community interaction—and excluding institutional or formal contexts—enables and maximizes cross-cultural comparability [33]. These samples yielded 1597 request and response sequences, with each of the languages in table 1 contributing a comparable number of cases (approx. 200).

**Table 1.** Languages and researchers involved in the study.

language	language family	location	researcher
Cha'palaa	Barbacoan	Ecuador	Simeon Floyd
English	Indo-European (Germanic)	United Kingdom	Kobin H. Kendrick
Italian	Indo-European (Romance)	Italy	Giovanni Rossi
Lao	Tai	Laos	N. J. Enfield
Murrinhpatha	Southern Daly	northern Australia	Joe Blythe
Polish	Indo-European (Slavic)	Poland	Jörg Zinken
Russian	Indo-European (Slavic)	Russia	Julija Baranova
Siwu	Kwa	Ghana	Mark Dingemanse

**Figure 2.**
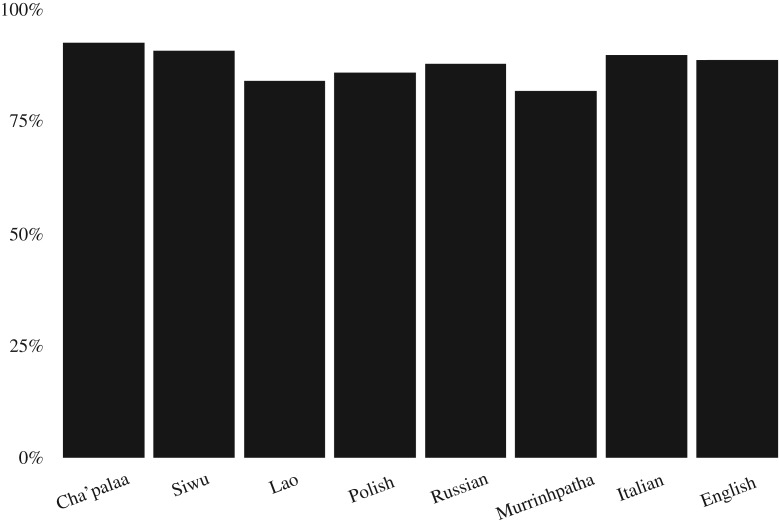
Frequency of fulfilment versus rejection in completed request sequences (1057 cases).

All cases of request and response sequences were exhaustively collected from stretches of informal interaction sampled from the respective corpora. These data provide evidence of a high degree of prosociality across cultures: of the 1057 cases in which there was an immediate, clear response to the request, only 129 cases were refusals to comply, while in the other 928 cases the requestee fulfilled the request. Figure 2 shows the breakdown of this set of cases in which request sequences were completed by either fulfilment or rejection of the request (excluding cases with other types of responses, e.g. asking for clarification, appearing not to hear, unclear response in the recording, etc.)

The overwhelming tendency towards fulfilment of requests was consistent across languages, showing a general, cross-cultural norm supporting assistance and collaboration. The cases in which the request was successful allow us to examine rates of expression of gratitude. Our criteria for identifying expressions of gratitude were functional: we included conventional phrases like ‘thank you’ as well as other forms that accomplish a similar social action like ‘good job’ or ‘sweet’, as illustrated in the following example from English.^1^

A: *Can I have one*? (looking at B's bag of biscuits)
B: *Yeah* (reaches into bag)
A: *Sweet*
[English. RCE06_878680a 0:14:39]

Many languages from smaller-scale communities have no set linguistic practices but can use other positive expressions as in the following example from the Australian language Murrinhpatha.

A: *Panguwangu nabattharra*
Take it over there
B: (takes object in direction indicated)
A: *Yukuy murruwurlnyima*
That's right, you're beautiful
[Murrinhpatha 20110828_GYHM100_03_541130]

We used a liberal definition of expressing gratitude, namely any positive conveyance of appreciation or satisfaction by the requester immediately after obtaining assistance or collaboration. Using these criteria, we coded each request sequence for whether it included an expression of gratitude. We then analysed the frequency of such expressions across our eight languages.

## 3. Material and methods: detailed

### 3.1. Project

The objective of this study was to examine the maintenance of social reciprocity by determining the extent to which people overtly express gratitude for the fulfilment of requests made in informal social interaction in a diverse sample of languages (table 1). This study is part of a larger project comparing the elements of request sequences (or recruitment sequences, more generally) in informal everyday interaction (http://recruitments.nickenfield.org). The first stage of the project consisted of data collection using methods of field linguistics and social interaction research to obtain audiovisual recordings of naturally occurring informal interaction *in situ* in the respective language communities.

### 3.2. Corpora

This study is based on the analysis of corpora of audiovisual recordings of informal everyday language usage in social interaction in eight languages from five continents. The construction of these corpora followed a similar procedure involving the placement of an unattended camera in household and community contexts, to record social interactions as they were occurring naturally, using high standards for audio and video quality. The data were then transcribed and translated by each language expert (table 1), typically with assistance from native speakers. The corpora range in size from about ten to over ninety hours of footage. In some cases, the corpus represents the largest available database for the language, especially in the case of unwritten minority languages like Cha'palaa, Murrinhpatha and Siwu. For larger-scale national languages like English, Italian, Lao, Polish and Russian, other corpora may be available to some degree, but most of these are limited to written language, due to the intensive demands of transcription of spoken language; demands which make corpus-based comparative studies like this one relatively new.

### 3.3. Sampling

After data collection, we applied a sampling procedure using methods of conversation analysis and interactional linguistics to identify request sequences (or recruitment sequences, defined more broadly). Researchers exhaustively identified all requests in samples of their corpora constructed so as to obtain a fair representation of settings and speakers and a similar number of cases for each language (approx. 200). Each request sequence minimally involved one person making a request for assistance or collaboration by doing or saying something to another so that they can perceive it (e.g. pointing to the salt or saying ‘Can you pass the salt?’), and a response by that person, typically involving the fulfilment of the request.^2^ The sequence could be further expanded by an expression of gratitude by the requester for the fulfilment.

### 3.4. Coding

Each request sequence in the dataset was coded by the respective language expert according to questions addressing both linguistic and interactional aspects of the sequence, allowing quantitative analysis and comparison. The design of this coding scheme was informed by extensive qualitative analysis and group discussion of request sequences in the languages. We determined that the coding scheme was consistently applied by all eight coders through a reliability check using a sub-sample of the English cases, which was independently second-coded by the other seven researchers. Coding questions that did not meet standards of reliability were either excluded from quantitative analysis or were recoded using a narrower coding instruction. The two specific questions focused on in this study were reliably answered for the categories considered (see below), achieving a Krippendorf's *α* [34] of greater than 0.75 and greater than 0.68, respectively.

The first question of relevance for the present study asked what type of response was provided to each request. Coders classified a response as fulfilment if the requestee immediately did the requested action or alternatively began to do it in cases where the nature of fulfilment did not allow for it to be quickly completed (e.g. washing dishes). A response was classified as rejection if the requestee overtly refused to do the requested action. Requestees could also respond in other ways, such as by ignoring the request or by asking for clarification or repetition of the request, or sometimes the outcome was not clear; coders classified these ‘other’ categories separately from ‘fulfilment’ and ‘rejection’.

(1) What is the response doing relative to the request?
  — fulfils or begins fulfilling
  — rejects
  — other (asks for clarification, ignores, unclear, etc.)

The second relevant question applied only to the subset of cases in which coders classified the response as ‘fulfilment’ in the previous question. These are the cases in which expression of gratitude is relevant.

(2) Is there an acknowledgement by the requester?
  — yes
  — no

Our criteria for identifying acknowledgement—another way to refer to expressing gratitude—were functional: we included conventional phrases like ‘thank you’ as well as other forms with a comparable effect like ‘good job’ or ‘sweet’; we also included explicit acknowledgements done non-verbally, for example by nodding one's head or making a hand gesture. Our definition of expressing gratitude was any positive conveyance of appreciation or satisfaction by the requester immediately after receiving a response indicating the fulfilment of the request.^3^ All identified expressions of gratitude with translation are reported in electronic supplementary material, table S1.

## 4. Analysis and results

**Figure 3.**
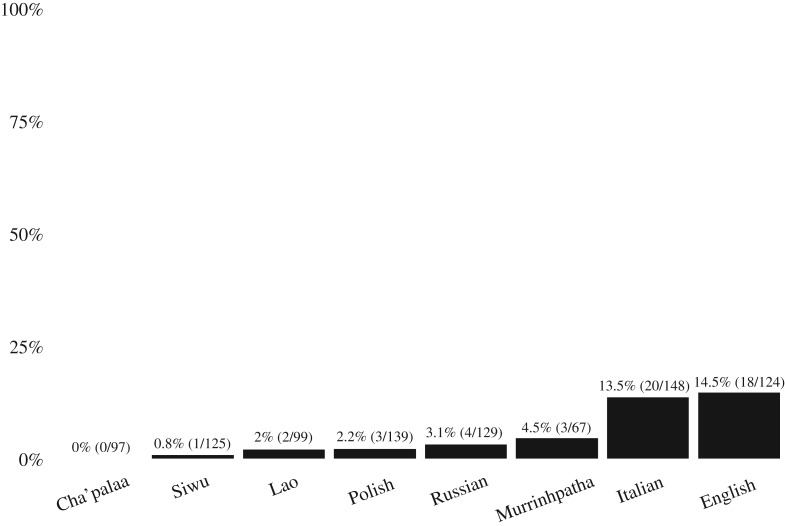
Frequency of expressions of gratitude after successful requests (928 cases).

Our findings are shown in figure 3. The main finding is that expressions of gratitude are very infrequent in all languages, occurring an average of just 5.5% of the time (*n* = 51/928). This indicates that in episodes of informal everyday interaction where gratitude is relevant, people seldom express it explicitly, suggesting that the maintenance of social reciprocity does not depend on the verbalization of gratitude.

While the expression of gratitude was rare across the board, there is minor but significant variation among languages, with the lowest frequency in Cha'palaa (0%, *n* = 0/96) and the highest in English (14.5%, *n* = 18/124). This lends support to the anecdotal evidence that the explicit expression of gratitude is more common in certain languages than in others. Two languages, English and Italian, have similar relatively higher rates of expressions of gratitude—although still far less often than one might expect based on the cultural ideology of politeness around thanking in Western cultures. In informal interaction between people who know each other well, even English speakers express gratitude just one out seven times when someone complies with a request.

To statistically probe the difference in frequency of expressing gratitude between the languages in our sample, we used mixed effects logistic regression with Siwu—showing the lowest rate among languages with at least one case of expression of gratitude—as a baseline, and with the rest of the languages ordered by relative frequency of gratitude expression (as shown in figure 3). The model included the recording from which each request sequence was taken as a random factor, with no significant effect. Table 2 reports the fixed effects of the model, indicating that Lao, Polish, Russian and Murrinhpatha are not statistically different from Siwu (*p* > 0.1), while both English and Italian are (English: OR 21.06, SE 1.04, *p* < 0.01; Italian: OR 19.38, SE 1.03, *p* < 0.01). This shows that speakers of English and Italian are more likely to express gratitude after having their requests fulfilled compared to speakers of other languages. That said, we emphasize that the English and Italian frequencies of expressing gratitude are still low, occurring at around one out of seven occasions.

**Table 2.** Fixed effects of mixed effects logistic regression, showing that speakers of English and Italian are more likely to express gratitude after a successful request than speakers of Siwu (intercept), whereas speakers of Lao, Polish, Russian and Murrinhpatha are not.

	estimate	s.e.	*z*-value	*p-*value
(intercept)	−4.8203	1.0038	−4.802	1.57 × 10^−6^
Lao	0.9491	1.2321	0.770	>0.1
Polish	1.0361	1.1612	0.892	>0.1
Russian	1.3783	1.1249	1.225	>0.1
Murrinhpatha	1.7600	1.1647	1.511	>0.1
Italian	2.9640	1.0322	2.872	<0.01
English	3.0472	1.0356	2.942	<0.01

## 5. Discussion and conclusion

In our data from everyday informal interaction across the world we find an abundance of episodes in which people successfully elicit another's provision of a good, service or support in the practicalities of everyday life around the home or village. The predominant success of requesters in having their requests fulfilled is not surprising given the highly cooperative nature of human sociality [1]. What is striking, however, is that most of these episodes culminate without the beneficiaries expressing gratitude. This suggests that people across languages and cultures rely on tacit understandings of their social rights and duties to mutual assistance and collaboration. One of the reasons for this is that, in everyday life, we are not just motivated to help or ‘do favours’ for others; we are also motivated to participate in shared activities that involve expected contributions, and to fulfil the commitments implied by our social roles; in other words, we are required to take and share in responsibility [35,36]. When someone's cooperation is expected as part of their contribution to the running of everyday affairs, it is not necessary to explicitly express gratitude on the spot. Gratitude for someone ‘doing their part’ will be experienced and sustained through someone else's reciprocal fulfilment of needs and responsibilities.

The results of this study indicate that care should be taken not to conflate the emotion of gratitude with the act of expressing it. Such expressions turn out to be very rare among friends, family and neighbours, whether in Africa, Asia, Australia, South America or Europe, and even among English speakers who place a special cultural value on saying ‘thank you’ as an important aspect of politeness. At the same time, the results give evidence of minor but significant cross-cultural variation in this respect. Speakers of English and Italian do seem to express gratitude more often than speakers of non-Western languages. For speakers of Lao (Southeast Asia) or Siwu (western Africa), saying ‘thank you’ is so rare that it may be perceived as bizarre or out of place, whereas English speakers in foreign contexts sometimes find it rude when gratitude is left unspoken. Languages like Cha'palaa (South America) have no conventional way to say ‘thank you’ at all, and while some speakers know the Spanish word ‘gracias’, they are unable to translate it. Although the artificial language Dothraki on the popular television show *Game of Thrones* is made out to be exotic for having ‘no word for thank you’, this is common in languages around the world, and when one exists, it tends to be a marked expression, and thus used with restraint. In English-speaking and other Western societies, expressions of gratitude for another's assistance or collaboration may occur primarily in institutional contexts and when interacting with strangers: a comparison of our findings on informal social interaction with others based on service encounters suggests that rates are higher in the latter [37,38].^4^

The low rates of expressions of gratitude seen cross-linguistically suggest that the global norm in everyday life is for gratitude to be left implicit and to be tacitly managed through the reciprocal fulfilment of social rights and duties. The slightly but significantly higher rates of expressing gratitude seen in English and Italian show that, as is often the case, speakers of English and other Western European languages turn out to be ‘outliers’ that are not representative of the diversity of the world's languages and cultures. Researchers should therefore use caution when coming to species-wide conclusions based on such populations [39]. Our results suggest that we must distinguish between a possibly universal *feeling* of gratitude and more culturally variable practices of *expressing* gratitude. Despite the attitudes encountered in some cultures that emphasize saying ‘thank you’ often, such practices do not appear to be necessary for the maintenance of everyday social reciprocity.

## Supplementary Material

Coded request sequences

## Supplementary Material

Expressions of gratitude with translation

## Footnotes

1 ‘Sweet’ here is not a comment on the biscuit's taste, as it precedes the actual tasting.

2 For reasons of comparability and analysability, we only included cases in which what is requested is an action to be performed here and now; requests for future actions were excluded.

3 A subsequent question (not reported here) asked if the requestee responded to the expression of gratitude with a phrase like ‘You're welcome’. This occurred in only three cases across the whole dataset.

4 Our analysis of request sequences included a number of linguistic and social-interactional variables that might potentially have an effect on gratitude expression, such as whether the request was verbal or non-verbal, or whether the requester had higher or lower status relative to the requestee. Such variables, however, did not have statistically significant effects on the general pattern we report. It is also worth noting that our focus on here-and-now requests implies that most of the actions requested are relatively straightforward and low-cost. The few cases of higher-cost requests we can note in our data are just too few to show an effect on gratitude expression; the investigation of such a potential effect is left to future research.

## Data accessibility

The datasets supporting this study have been uploaded as part of the electronic supplementary material.
